# Personalized neurorehabilitative precision medicine: from data to therapies (MWKNeuroReha) – a multi-centre prospective observational clinical trial to predict long-term outcome of patients with acute motor stroke

**DOI:** 10.1186/s12883-022-02759-2

**Published:** 2022-06-30

**Authors:** Corinna Blum, David Baur, Lars-Christian Achauer, Philipp Berens, Stephanie Biergans, Michael Erb, Volker Hömberg, Ziwei Huang, Oliver Kohlbacher, Joachim Liepert, Tobias Lindig, Gabriele Lohmann, Jakob H. Macke, Jörg Römhild, Christine Rösinger-Hein, Brigitte Zrenner, Ulf Ziemann

**Affiliations:** 1 Department for Neurology & Stroke, University Hospital of Tübingen, Hoppe-Seyler-Str. 3, 72076 Tübingen, Germany; 2 Hertie Institute for Clinical Brain Research, Ottfried-Müller-Straße 25, 72076 Tübingen, Germany; 3 medical Data Integration Centre (meDIC), University Hospital of Tübingen, Schaffhausenstr. 77, 72072 Tübingen, Germany; 4 University Hospital of Tübingen, Institute for Ophthalmic Research, Elfriede-Aulhorn-Str. 7, 72076 Tübingen, Germany; 5 Cluster of Excellence Machine Learning, University of Tübingen, Maria-von-Linden-Str. 6, 72076 Tübingen, Germany; 6 Department for Biomedical Magnetic Resonance, University Hospital of Tübingen, Ottfried-Müller-Str. 51, 72076 Tübingen, Germany; 7 Max Planck Institute for Biological Cybernetics, Max-Planck-Ring 8-14, 72076 Tübingen, Germany; 8 SRH Gesundheitszentrum Bad Wimpfen GmbH, Bei der alten Saline 2, 74206 Bad Wimpfen, Germany; 9 University hospital of Tübingen, Institute for translational Bioinformation (TBI), Schaffhausenstr. 77, 72072 Tübingen, Germany; 10 University of Tübingen, Interfaculty Institute for Biomedical Informatics (IBMI), Sand 14, 72076 Tübingen, Germany; 11 Department of Computer Science, Applied Bioinformatics (ABI), University of Tübingen, Sand 14, 72076 Tübingen, Germany; 12 Schmieder Clinic Allensbach, Zum Tafelholz 8, 78476 Allensbach, Germany; 13 Department for Diagnostic and Interventional Neuroradiology, University Hospital of Tübingen, Hoppe-Seyler-Str. 3, 72076 Tübingen, Germany; 14 Department for High-field Magnetic Resonance, Max Planck Institute for Biological Cybernetics, Max-Planck-Ring 11, 72076 Tübingen, Germany

**Keywords:** Motor outcome, Outcome prediction, Acute stroke, Upper extremity, Personalized neurorehabilitation, Machine learning

## Abstract

**Background:**

Stroke is one of the most frequent diseases, and half of the stroke survivors are left with permanent impairment. Prediction of individual outcome is still difficult. Many but not all patients with stroke improve by approximately 1.7 times the initial impairment, that has been termed proportional recovery rule. The present study aims at identifying factors predicting motor outcome after stroke more accurately than before, and observe associations of rehabilitation treatment with outcome.

**Methods:**

The study is designed as a multi-centre prospective clinical observational trial. An extensive primary data set of clinical, neuroimaging, electrophysiological, and laboratory data will be collected within 96 h of stroke onset from patients with relevant upper extremity deficit, as indexed by a Fugl-Meyer-Upper Extremity (FM-UE) score ≤ 50. At least 200 patients will be recruited. Clinical scores will include the FM-UE score (range 0–66, unimpaired function is indicated by a score of 66), Action Research Arm Test, modified Rankin Scale, Barthel Index and Stroke-Specific Quality of Life Scale. Follow-up clinical scores and applied types and amount of rehabilitation treatment will be documented in the rehabilitation hospitals. Final follow-up clinical scoring will be performed 90 days after the stroke event. The primary endpoint is the change in FM-UE defined as 90 days FM-UE minus initial FM-UE, divided by initial FM-UE impairment. Changes in the other clinical scores serve as secondary endpoints. Machine learning methods will be employed to analyze the data and predict primary and secondary endpoints based on the primary data set and the different rehabilitation treatments.

**Discussion:**

If successful, outcome and relation to rehabilitation treatment in patients with acute motor stroke will be predictable more reliably than currently possible, leading to personalized neurorehabilitation. An important regulatory aspect of this trial is the first-time implementation of systematic patient data transfer between emergency and rehabilitation hospitals, which are divided institutions in Germany.

**Trial registration:**

This study was registered at ClinicalTrials.gov (NCT04688970) on 30 December 2020.

## Background

Stroke is one of the most common neurological diseases, which leaves one third of patients dead and one third with lasting disabilities despite the best standard of medical care [1]. Up to 70% of all acute stroke patients present with paresis of one upper extremity (UE) [2].

Recovery mostly occurs in the first weeks and plateaus at approximately three months after stroke. Up to 70% of recovery occurs spontaneously as a result of a sensitive phase of enhanced neuroplasticity directly after stroke, independent of rehabilitative interventions [3]. Rehabilitative interventions try to make use of this sensitive period by starting as early as possible while the patient is still on the stroke unit (SU). However, fixed rehabilitation procedures are usually applied, largely irrespective of stroke type (e. g., lacunar vs. territorial infarction, ischemic vs. hemorrhagic stroke), location (e. g., cortical vs. subcortical), or severity. It is therefore not surprising that some patients benefit from rehabilitation treatment more than others. Outcome varies depending on volume and localization of stroke lesion, clinical severity of stroke and integrity of the corticospinal tract (CST) [4–6]. For instance, a lesion in the posterior limb of the internal capsule is associated with poorer outcome than a lesion in the primary motor cortex [7]. Preserved interhemispheric functional and effective connectivity between the primary motor cortex (M1) and the supplementary area (SMA) are predictive for better motor outcome [8, 9]. Slow oscillations in resting-state electroencephalography (EEG) and low-complexity high-amplitude transcranial magnetic stimulation (TMS)-evoked EEG responses (TEPs) are associated with poor overall outcome [10–12].

Acute stroke severity is routinely assessed by the National Institute of Health Stroke Scale (NIHSS, range 0–42, 0 indicates absence of a neurological deficit) [13]. The initial NIHSS is related to stroke outcome. For instance, an NIHSS < 3 predicts an excellent outcome after three months, while a score > 15 is associated with poor outcome [6]. There is mounting evidence that inflammation during the acute phase after stroke influences severity and outcome. Elevation of C-reactive protein (CRP), for example, is associated with poor long-term outcome [14, 15]. Other blood biomarkers potentially relevant for stroke outcome are electrolytes, d-dimers, N-terminal proBrain natriuretic peptide (NT-proBNP) and troponin [14, 16, 17].

In addition, the integrity of the corticospinal tract (CST) is crucial for motor outcome. Integrity of CST can be evaluated by motor evoked potentials (MEPs) which is indeed one of the most robust biomarkers for motor outcome in stroke [4, 18].

Despite years of research and clinical practice, up to date it is neither possible to predict motor outcome after stroke reliably, nor which rehabilitation treatment to select for optimal outcome [3], although there were several attempts: For example, Stinear and colleagues [4] created an algorithm (Predict Recovery Potential 2 (PREP 2)), that included testing of shoulder abduction and finger extension (SAFE), patient age as well as presence or absence of MEPs, that determined UE outcome correctly for 75% of their cases, while 25% were still incorrectly classified. Other studies demonstrated that most patients recover by approximately 70% compared to their initial impairment, which has been termed the proportional recovery rule [19]. However, not all patients behave in accord with this rule, a phenomenon that has not been fully elucidated [20].

The present study aims not only to close this knowledge gap but also strives to observe to what extent type and amount of rehabilitation treatment relates to improvement of hand/arm motor function in stroke.

Towards this end, we will collect an extensive set of clinical, electrophysiological, imaging and laboratory data during the acute phase of stroke, and dense clinical follow-up data during rehabilitation and 90 days after stroke. Furthermore, type and amount of applied rehabilitation treatment will be recorded. The complex data will be analyzed by machine learning algorithms to identify predictive patterns for favorable stroke outcome. This may eventually help defining personalized rehabilitation, i.e., which patient benefits best from which therapy, and to what extent.

## Methods

### Objectives

The objective of the present study is to identify factors predicting motor outcome after stroke more accurately than hitherto possible, and rehabilitation treatments that are associated with improvement of arm−/hand function in individual motor stroke patients, using machine learning algorithms.

Primary objective: To determine which factors of the primary data set within 96 h of stroke onset and which type and amount of rehabilitation treatment predict the primary endpoint, i.e., the change in the FM-UE defined as 90 days FM-UE minus initial FM-UE, divided by initial FM-UE impairment.

Secondary objectives: Secondary endpoints are the quality of life, independence and range of activity of the UE measured by stroke-specific quality of life (SS-QOL) scale, modified Rankin Scale (mRS), Barthel-Index (BI) and Action Research Arm Test (ARAT) 90 days after the stroke event compared to the initial scores in the acute phase.

### Trial design

MWKNeuroReha is designed as a multi-centre prospective observational clinical trial. The framework is exploratory. The study was registered at ClinicalTrials.gov (NCT04688970, registry name: Personalized Neurorehabilitative Precision Medicine – From Data to Therapies) on 30 December 2020 and is expected to completed 31 December 2022.

For more administrative information see Table 1.

**Table 1:** Administrative information

Title	Personalized neurorehabilitative precision medicine: from data to therapies (MWKNeuroReha) – a multi-centre prospective observational clinical trial to predict long-term outcome of acute stroke patients with acute motor stroke	
Trial registration	ClinicalTrials.gov Identifier: NCT04688970 Registry name: Personalized Neurorehabilitative Precision Medicine – From Data to Therapies Date of registration: 30 December 2020 Secondary identifying numbers: BNP-2020-09 Contact for public and scientific queries: Corinna Blum and David Baur, University Hospital of Tübingen, Department for Neurology & Stroke and Hertie Institute for Clinical Brain Research; phone: 0049–7071–29-61,788; e-mail: neuroreha@med.uni-tuebingen.de Countries of recruitment: Germany Health condition studied: acute stroke with affection of the UE Interventions and study type: observational study (no interventions) Key inclusion and exclusion criteria: • Ages eligible for study: ≥18 years • Sexes eligible for study: both • Accepts healthy volunteers: no • Main inclusion criterion: acute motor stroke with functional relevant deficit of the UE • Main exclusion criteria: no acute stroke, no relevant affection of UE, contraindication to MEPs Date of first enrolment: 01 December 2020 Target sample size: 200 patients Recruitment status: recruiting Primary outcome: percentage change in the FM-UE defined as 90 days FM-UE minus initial FM-UE, divided by initial FM-UE impairment: $\frac{\mathrm{FMUE}\left(90\ \mathrm{days}\right)-\mathrm{FMUE}\left(\mathrm{initial}\right)}{66-\mathrm{FMUE}\left(\mathrm{initial}\right)}\times$ Secondary outcome: • Action research arm test (ARAT) [21]: to measure recovery of function of the UE • Modified Rankin scale (mRS) [22]: to measure neurological impairment • Barthel index (BI) [23]: to measure performance in activities of daily living • SS-QOL scale [24]: to measure quality of life and	
Protocol version	Issue dates: 21 Sep 2020 (original protocol) / 23 Nov 2020 (protocol amendment 1) Protocol amendment Number: 01 Authors: CB, UZ	
	2020-Sep-21	Original
	2020-Nov-23	Amendment 01: Primary reason for amendment: Changes in section 12. Clarification of how to handle patients not able to give informed consent Additional changes: More detailed description of safety precautions for transcranial magnetic stimulation, more detailed description of data exchange between study sites, revised patient information and data protection declaration
	2021-Jan-12	Amendment 02: Inclusion of additional cooperating hospital sites.
	2021-Mar-21	Amendment 03: Implementation of formal changes in the consent form demanded by a subordinated ethic committee responsible for a cooperating hospital.
Funding	The study is funded by the Department of Science and Arts of the federal state of Baden-Württemberg, Germany.	
Author details	UZ developed the study design, and obtained the funding. DB, BZ, FF and JL helped with implementation under the coordination of CB and UZ. CB and CRH acquired the first data sets. OK, SB, JR and LCA built and maintain the data bank. ME, TL and GL provided expertise for acquiring and analyzing imaging data. PB, JHM, ZH and LCA provided expertise for data analysis through machine learning algorithms.	
Name and contact information for the trial sponsor	Hertie Institute for Clinical Brain Research Ottfried-Müller-Straße 25, 72,076 Tübingen, Germany Executive Officer: Dr. Astrid Proksch Phone: + 49–7071-2,987,641 E-Mail: astrid.proksch@medizin.uni-tuebingen.de	
Role of sponsor	Study sponsor and funder had no role in the design of this study and will not play any role during its execution, management, collection, analysis or interpretation of the data, writing of the report, or decision to submit the report for publication. Study sponsor and funder will not have ultimate authority over any of these activities.	

Abbreviations: *UE* upper extremity, *MEPs* motor evoked potentials, *FM-UE* Fugl-Meyer-upper extremity-score, *ARAT* Action Research Arm Test, *mRS* modified Rankin Scale, *BI* Barthel Index, *SS-QOL* Stroke Specific Quality Of Life Scale

### Participants, interventions and outcomes

#### Study setting

The study will take place in the University Hospital of Tübingen (southwest Germany). In this tertiary referral hospital > 1000 acute stroke patients are treated every year. The University Hospital of Tübingen is the lead partner of the Centre for Neurovascular Diseases, a network of seven cooperating acute-care hospitals to provide optimal treatment for stroke and other neurovascular diseases. Four of these hospitals will act as additional study sites for recruitment of acute stroke patients. The acute-care hospitals collaborate with seven neurorehabilitation hospitals where the acute stroke patients of this study are transferred to for rehabilitation treatment.

#### Eligibility criteria

Inclusion criteria:

- 18 years or above
- Acute motor stroke with functionally relevant UE deficit (FM-UE ≤ 50)
- Participant understands the study and its procedures and provides written informed consent
- If the participant is not able to provide informed consent:
  ∘ The assumed will of the patient is assessed by the participant’s provision (if existing), the health care proxy (if existing) and/or the will that has been expressed by the patient to close relatives
  ∘ The legal representative provides written informed consent based on this assessment

Exclusion criteria:

- Less than 18 years old
- No acute stroke, or stroke does not affect UE function, or FM-UE > 50
- Participant or legal representative do not provide written informed consent
- Participant has an intracranial implant (e.g., aneurysm clips, shunts, stimulators, cochlear implants, or electrodes) or any other metal object within or near the head (excluding the mouth) that cannot be safely removed
- Participant has a history of any illness that, in the opinion of the study investigator, might confound the results of the study
- Any concern by the investigator regarding the safe participation of the participant in the study, or any other reason because of which the investigator considers the participant inappropriate for study inclusion
- Pregnancy
- Participant refuses to receive neurorehabilitation treatment

Besides, a blood sample (10 ml) will be obtained and stored in the local biobank of the Medical Faculty of the University of Tübingen/University Hospital of Tübingen for possible further retrospective analyses. Extra written informed consent will be obtained from each participant.

Medical doctors will screen the patients for participation, explain the study to them, answer their questions and finally obtain their written informed consent, provided the patients or their representatives understand the purpose and procedures of the study, are able to provide written informed consent and are willing to participate.

### Outcomes

The primary endpoint is the percentage change in the FM-UE defined as 90 days FM-UE minus initial FM-UE, divided by initial FM-UE impairment:

$$\frac{\mathrm{FMUE}\left(90\ \mathrm{days}\right)-\mathrm{FMUE}\left(\mathrm{initial}\right)}{66-\mathrm{FMUE}\left(\mathrm{initial}\right)}\times 100\%$$

The FM-UE [25] is a highly standardized, reliable and validated clinical score to assess deficits in upper extremity function after stroke [26]. The primary endpoint will allow to measure to what extent a given participant will deviate from the predicted proportional recovery rule of 70% improvement of the initial FM-UE impairment [19].

Secondary endpoints are the changes in the following tests 90 days after the stroke event compared to the initial test results within 96 h after stroke onset:

- Action research arm test (ARAT) [21]: to measure recovery of function of the UE
- Modified Rankin scale (mRS) [22]: to measure neurological impairment
- Barthel index (BI) [23]: to measure performance in activities of daily living
- SS-QOL scale [24]: to measure quality of life and demonstrate the influence of the scores described above on the personal experience of the patient

All of these tests have shown reliability and have been validated in the assessment of patients post-stroke [22, 24, 27, 28].

### Participant timeline

In the first 96 h after stroke onset the primary data set (see below) is acquired by the University Hospital of Tübingen, or one of the other cooperating acute-care hospitals.

0–24 h: The patients will be screened for participation, written informed consent will be obtained, the first clinical tests will be conducted adapted to the patient’s fitness, and a blood sample (part of the usual stroke workup) is collected.

25–48 h: Neuroimaging (magnetic resonance imaging (MRI) or computed tomography (CT)) will be conducted and further clinical tests adapted to the patient’s fitness will be performed.

49–96 h: Adapted to the patient’s fitness, clinical tests, EEG and motor evoked potentials (MEPs) will be performed.

After completion of acute stroke treatment, participants are transferred to collaborating neurorehabilitation facilities. There, the rehabilitation data set is collected:

On admission: FM-UE, BI and mRS.

During the stay:

- At intervals of 14 days: FM-UE, BI and mRS
- number, duration and type of neurorehabilitative treatments

On discharge:

- FM-UE, BI and mRS

Final examination:

After 90 days the final follow-up examination is carried out. The following data is collected: FM-UE (primary endpoint), ARAT, BI, mRS, SS-QOL scale (secondary endpoints).

There are no interventions planned in this observational study. All participants are treated according to the current guidelines, the same way they would have been treated outside of this study.

### Sample size

We will use machine learning algorithms to predict the primary end point (i.e., the change in FM-UE 90 days after the stroke event compared to the initial FM-UE, see definition under “Outcomes”) as a function of the primary data set and the rehabilitation data set detailed in Tables 2, 3. Model development will start once 50 patients have been reached and continue throughout the study. To cope with the dimensionality of the problem, we will use lasso-regularized linear regression to select a sparse subset of the potentially correlated features. We will use a nested-cross validation setup to tune the regularization strength via the 1SE-rule [29] and obtain estimates of the fraction of the explained variance of the total variance in the primary end point on data not seen during model training. Due to the exploratory nature of the project and the complex models involved, a precise sample size calculation is not feasible.

**Table 2:** Timeline for collecting the primary data set in the university hospital and other study sites

		Clinical data	Imaging data	Electrophysiological data	Laboratory data
0–24 h		NIHSS*			Blood sample*
25–48 h		mRS*	MRI* or CT*		
49–96 h		SAFE score		EEG	
				MEP	
		Grip strength			
		FMA			
		ARAT			
		BI*			
		Bells test			
		AST			
		BDI			
		SS-QOL scale			

Tests marked with * are part of the usual stroke work-up

Abbreviations: *NIHSS* National Institute of Health Stroke Scale, *mRS* modified Rankin Scale, *SAFE* Shoulder Abduction Finger Extension score, *FMA* Fugl-Meyer Assessment, *ARAT* Action Research Arm Test, *BI* Barthel Index, *MRI* magnetic resonance imaging, *CT* computed tomography, *Aphasie-Schnelltest, AST* aphasia quick test, *BDI* Beck’s Depression Inventory, *SS-QOL* Stroke-Specific Quality Of Life scale, *EEG* electroencephalography, *MEP* motor evoked potential

**Table 3:** Overview over complete data collection

Location	Time point	Data set
University hospital of Tübingen, other recruiting study sites	Day 1–4 after stroke	Primary data set (see Table 1)
Rehabilitation hospital	On admission	Rehabilitation data set: FM-UE, BI and mRS
	Every 14 days during the stay and on discharge	• FM-UE, BI, mRS and questionnaire to capture soft influencing factors • Number, duration and type of neurorehabilitative treatments
University hospital	Day 90 after stroke	FM-UE, ARAT, BI, mRS, SS-QOL scale

Abbreviations: *FM-UE* Fugl-Meyer upper extremity score, *BI* Barthel Index, *mRS* modified Rankin Scale, *ARAT* Action Research Arm Test, *SS-QOL* Stroke-Specific Quality Of Life scale

### Recruitment

All acute stroke patients (> 2000 per year in the University Hospital of Tübingen and the 4 cooperating acute-care hospitals of the Center of Neurovascular Diseases) will be screened for recruitment, which is planned for a duration of 1.5 years. Up to 70% of all stroke patients present with UE paresis [2, 30], thus about 2000 patients (2000 patients /year × 1.5 years (recruitment period) × 0,7 (percentage of patients presenting with affection of UE)) are available for recruitment in theory. In practice (according to a preparatory analysis of our stroke unit data base over the preceding 2 years), approximately 60% of these patients will not have a sufficient deficit defined as FM-UE ≤ 50 initially or they will have a sufficient deficit, but it will not lasting beyond 96 h resulting in the patient not being in need of rehabilitation of the UE and not eligible for the project anymore (since he or she is not meeting the inclusion criteria anymore), or will die or be too frail for rehabilitation treatment, or will not fulfil the inclusion/exclusion criteria to begin with. Out of the remaining group of 800 patients there will be an expected further attrition by approximately 50% due to unwillingness to participate in the study or failure to obtain written informed consent, leaving approximately 400 patients for recruitment (i.e., a safety margin of 100 patients given the planned inclusion of 300 patients).

Unfortunately, the recruitment process showed that despite the conservative calculation we will probably only reach 200 patients.

### Data collection and management

#### Plans for assessment and collection of outcomes

Each participant will be assessed carefully within the first 96 h after stroke onset in the acute-care hospitals, using an extensive battery of standardized and validated clinical tests and scores. Additionally, laboratory, neuroimaging and electrophysiological data will be obtained. These data constitute the primary data set. In the rehabilitation hospitals some of the clinical scores of the primary data set will be obtained repeatedly at 14 days intervals, and the amount, type and intensity of neurorehabilitation treatment will be documented. These data constitute the neurorehabilitation data set. All data will be managed and integrated in a customized secure clinical data capture system (REDCap®, Research Electronic Data Capture) to which some of the data will be transferred automatically from primary clinical data information systems, while other data will be inserted manually.

All of the following clinical tests are conducted by or under the supervision of a qualified medical doctor of the University Hospital of Tübingen (Department of Neurology), a collaborating acute-care hospital of the Centre of Neurovascular Diseases, or a cooperating rehabilitation facility. Diagnostic neuroimaging is performed by a qualified medical doctor of the department of neuroradiology of the University Hospital of Tübingen or a trained medical doctor of the collaborating hospitals. All electrophysiological tests are performed by or under the supervision of a qualified medical doctor of the University Hospital of Tübingen, department of neurology, or of the collaborating hospitals.

##### The primary data set

Clinical tests and scores:

The following clinical tests were chosen to describe the subject’s clinical condition as precisely as possible. The tests include measures for the impairment of UE function, which is the main focus of the study, but also other stroke symptoms, such as aphasia and neglect and other conditions (e.g., depression) that might affect the rehabilitation process. In the following section each test is described in detail:

National Institutes of Health Stroke Scale (NIHSS): The NIHSS is part of the usual highly standardized stroke workup. It consists of 15 items that can be scored with 0 to a maximum of 2–4 points. The score ranges between 0 and 42 points, with 0 indicating no deficit and 42 the maximum possible deficit. It is used to measure stroke severity and suitable to detect improvement or deterioration of the patient. The NIHSS has a high reliability (κ = 0.77) and validity (high correlation with CT lesion and clinical outcome, with Spearman’s correlations of 0.74 and 0.71, respectively) [13].

FM-UE and sensation: The FM-UE and sensation describes the sensorimotor impairment of the arm after stroke. It consists of 33 items for motor function scored from 0 to 2. A high score corresponds to high function. Reliability and validity is high, especially for motor function [26, 31]. The FM-UE is not part of the usual stroke workup. The change of the FM-UE after 90 days compared to the initial score obtained on the stroke unit during the acute phase after stroke related to the initial FM-UE impairment (for specific definition, see “Outcome”) will serve as the primary endpoint of this study. In addition, the FM-UE for sensation (6 items with scores 0–2) will be additionally assessed, but only for the primary data set. It will not serve as an endpoint of this study.

Shoulder Abduction Finger Extension (SAFE) score: To calculate the SAFE score, shoulder abduction and finger extension strength are measured using the classification of the British Medical Research Council (MRC). The MRC scale scores muscle strength from 0 (no movement) to 5 (normal power) [32]. Accordingly, the SAFE score ranges from 0 to 10. A score of 5 or more predicts a good or excellent outcome after stroke affecting the UE [4]. It is not part of the usual stroke workup.

Grip strength: The grip strength can be quantified using a dynamometer and is given in kilogram. The best out of three trials will be evaluated. It is not part of the usual stroke workup.

Bells test [33]: The Bells test assesses neglect by requesting the subjects to cross all the bells (*n* = 35) mixed with distractors (*n* = 280) on a sheet of paper. Missing out 5 bells ore more counts as evidence for neglect [34]. The Bells test shows a low learning effect and, therefore, can be used for repeated testing. It is not part of the usual stroke workup.

Aphasia Quick Test (German: Aphasie-Schnelltest) (AST): The AST is a short test for patients with acute aphasia inspecting comprehension, talking, reading and writing. It is scored from 0 to 31, a low score reflects severe aphasia. Validity and reliability is moderate to good (Kendall correlation coefficient 0.75–0.82 and Spearman’s correlation 0.68–0.82, respectively) [22, 35]. It is not part of the usual stroke workup.

mRS: The mRS is a widely used test to determine impairment and dependency after stroke on a scale ranging from 0 (no symptoms) over 1 (symptoms but no disability), 2 (slight disability), 3 (requires help, but can walk without assistance), 4 (cannot walk without assistance), 5 (bedridden, severe disability, requires constant nursing) to 6 (death). Reliability is moderate to good. Validity measured by correlation with infarct volume is moderate. This is comparable to other scales like the BI [6]. It is part of the usual stroke workup.

BI: The BI measures performance in activities daily life. Ten items can be scored from 0 to 5–15 points maximum, adding up from 0 to 100 points. A high score reflects high independence. Reliability is high [27], validity is moderate (measured by correlation with infarct volume) [6]. The BI is part of the usual stroke workup.

ARAT: The ARAT assesses motor activity performance of the UE after stroke. It consists of the subscales grasp, grip, pinch and gross movements which are scored from 0 (no movement) over 1 (movement only partially possible), 2 (movement possible but only with great difficulty or much time is needed) to 3 (normal movement), adding up to 57 points maximum. A score less than 10 points reflects severe impairment. Reliability is high (Cronbach’s alpha 0.97), and validity is moderate to good (ρ = 0.45–0.76) [36]. It is not part of the usual stroke workup.

SS-QOL scale: The SS-QOL scale measures health related quality of life. It consists of 49 items that are scored from 1 to 5, adding up to 49–245 points. A high score reflects high quality of life. Reliability is high (Cronbach’s alpha 0.73–0.89) with excellent content validity [24]. It is not part of the usual stroke workup.

Beck’s Depression Inventory (BDI): The BDI is a depression screening tool consisting of 21 items that are scored from 0 to 3, adding up to 0 to 63 points. A high score reflects high probability of depression, the threshold for the diagnosis of depression is 10. The BDI is reliable, content and convergent validity in particular is high, while differentiating from anxiety is poor [37]. It is not part of the usual stroke workup.

Diagnostic Instrument for Limb Apraxia (short version) (DILA-S): The DILA-S consists of 6 subscales: I. Imitation of meaningless gestures, II. Familiar Tool Test, III. Pantomime the usage of tools, IV. Imitation of meaningful gestures, V. Novel Tools and VI. Naturalistic Action Test – Breakfast Task [38]. We will only use subscale I and III.

I. Imitation of meaningless gestures: This subscale consists of 10 items. Each item is demonstrated by the examiner and has to be repeated by the subject with the non-impaired limb. The items are scored from 2 (correct imitation at the first try) over 1 (correct imitation at the second try) to 0 (false imitation). Consequently, a low score reflects severe apraxia. Interrater-reliability is high (97% congruence) and validity sufficient (r ≥ 3.39, *p* ≤ 0.003) [38]. It is not part of the usual stroke workup.

III. Pantomime the usage of tools: This subscale contains 8 items. Every item is a tool, for instance a key, that is presented on a picture and explained to the patient by the examiner. After demonstration the subject is asked to mimic the usage of the tool (e. g., open the door with a key) using the non-impaired limb. Each item is scored from 2 (correct at the first try), over 1 (correct at the second try) to 0 (total error). Hence, a low score corresponds to severe apraxia. Interrater-reliability is high (95% congruence) and validity sufficient (r ≥ 3.39, *p* ≤ 0.003) [37]. It is not part of the usual stroke workup.

In addition to these clinical tests and scores, other relevant clinical data (e. g., vital parameters, medication) will be collected. In the University Hospital of Tübingen these data will be retrieved semi-automatically from the primary clinical data information systems, while it will be inserted manually at the collaborating acute-care hospitals.

Laboratory workup:

Routine laboratory workup as part of the usual stroke workup will be collected with attention to sodium, calcium, CRP, d-dimers, NT-proBNP, troponin, international normalized ratio (INR) and activated partial thromboplastin time (aPTT). The selection of parameters is based on demonstrated correlations with stroke outcome [14–17, 39].

Neuroimaging:

For each participant structural neuroimaging will be acquired. Structural magnetic resonance imaging (MRI) comprises diffusion weighted imaging (DWI), fluid attenuated inversion recovery (FLAIR) and magnetic prepared-rapid gradient echo (MP-RAGE) sequences. Structural MRI data will be acquired at a 1.5 or 3 Tesla MRI scanner. Before scanning, patients are evaluated by a medical doctor for MRI contraindications. If MRI is not available or contraindicated, a cranial computed tomography (CT) will be performed. MRI/CT is part of the usual stroke work-up. There will be no additional study-related scanning beyond the clinical standard.

The images will be used to calculate lesion volume, and check for involvement of the pyramidal tract and other strategic locations influencing motor outcome [7]. The strategic locations are the primary motor cortex (M1), the corona radiata (with or without affecting the pyramidal tract), internal capsule (with or without affecting the pyramidal tract), corpus callosum, lentiform nucleus (putamen and globus pallidus), caudate nucleus, thalamus, crus cerebri (with or without affecting the pyramidal tract), mesencephalon (with or without affecting the pyramidal tract), pons (with or without affecting the pyramidal tract), medulla oblongata (with or without affecting the pyramidal tract), upper, middle or lower cerebellar peduncle.

Electrophysiological data:

EEG: At the University Hospital of Tübingen resting-state EEG will be obtained using a 21-channel (in patients with severe stroke) or 64-channel (in patients with non-severe stroke, who tolerate a longer procedure) gel filled sintered ring electrode EEG cap (EasyCap, Munich, Germany) and an optically isolated battery powered biosignal amplifier (Brain Products, Gilching, Germany). EEG will be recorded with eyes closed and eyes open for 3 min each. The other acute-care study centers will use a 21-channel EEG (TrueScan (DEYMED Diagnostic GmbH, Weimar, Germany), Natus Brain Monitor (Natus Europe GmbH, Planegg, Germany), EEG-40 T or EEG-29 T (Monitor (Natus Europe GmbH, Planegg, Germany), respectively).

The resting-state EEG will be analyzed using the pairwise devised Brain Symmetry Index (pdBSI) and the (delta+theta)/(alpha+beta) power ratio (DTABR). The BSI (or pdBSI if the data is assessed from homologue channels from both hemispheres) describes the ipsilesional/contralesional power (a)symmetry by quantifying the difference in mean spectral power per hemisphere across 1–25 Hz [11, 12]. As the term DTABR already implicates, slow (delta and theta) and fast (alpha and beta) brain oscillations are put in relation to each other. The DTABR and the pdBSI predict impairment as rated by the modified Rankin Scale (mRS) 6 months after the stroke event [11, 12].

Motor evoked potentials: MEPs are elicited by TMS and recorded by surface EMG. TMS is a technique that evokes action potentials in the cortex with a spatiotemporal precision of millimeters and milliseconds. In the primary study center a conventional TMS stimulator (MagPro Compact, MagVenture GmbH, Willich, Germany) and stimulation coil (C-B60, MagVenture GmbH, Willich, Germany) will be used. Surface EMG will be obtained through an optically isolated battery powered biosignal amplifier (Brain Products, Gilching, Germany) using bipolar electrodes from arm and hand muscles (extensor carpi radialis (ECR) and first dorsal interosseous (FDI) muscle) on the paretic and non-affected sides. In the collaborating study centers the DuoMAG MP (DEYMED Diagnostic GmbH, Weimar, Germany) and Magstim 200^2^ (ANT Neuro, Netherlands) magnetic stimulators will be used.

MEPs are measured with pre-innervation of the target muscle or, if not possible, the contralateral homologue muscle and maximum stimulator output (if required) to determine if the patient is MEP- or MEP+. The outcome MEP+ requires at least 50 μV peak-to-peak amplitude MEPs in the target muscle in at least 5 out of 10 consecutive trials. MEPs are one of the most researched and robust biomarkers in stroke outcome prediction. Positive predictive value (PPV) (if the patient is MEP+) is 1.0, while the negative predictive value (NPV) (if the patient is MEP-) is 0.74 [18].

##### The neurorehabilitation data set

In the rehabilitation facilities number and duration of therapies will be documented and classified according to the type of neurorehabilitative training. The rehabilitative treatments will be reported using the remedy position number. This is a number allocated to each rehabilitative treatment to make them transparent to the cost centers, i.e., the health care or pension insurance. To ensure that these interventions do not differ between the participating rehabilitation hospitals, they will be checked for complete congruence across hospitals.

In addition, therapy-influencing co-factors, namely support by relatives, motivation, additional self-training and relationship between patient and therapist are registered using a questionnaire with a Likert scale from 0 to 3 (never/very poor to daily/very good). This questionnaire is filled in by the primary therapist (usually a physiotherapist or an occupational therapist). Furthermore, intercurrent diseases during the stay in the rehabilitation facility possibly compromising the rehabilitation process will be registered.

In addition to the rehabilitation training, the FM-UE, the mRS and the BI will be documented on admission, in intervals of 14 days and on discharge.

### Plans to promote participant retention and complete follow-up

The data will be collected on the stroke units of the collaborating acute-care hospitals (primary data set) and the rehabilitation hospitals (neurorehabilitation data set) in a clinical setting defined by current guidelines for stroke care and neurorehabilitation. It is, therefore, not expected that patients discontinue their participation in this study. Only the final examination 90 days after the stroke event will be performed, in most cases, outside of clinical treatment. It will take place in the University Hospital of Tübingen or in the rehabilitation hospital, if the participant is still in rehabilitative treatment. If a participant is unable to come to the University Hospital of Tübingen for the final examination, he will be visited at home by members of the study team.

### Data management

All data of this study will be centrally stored in a clinical data capture system (REDCap®) [39, 40] operated in a secure environment by the Medical Data Integration Centre (meDIC) of the University Hospital Tübingen. Data will be inserted manually or transferred automatically from primary clinical information systems as available, and finally manually curated in the REDCap® data bank. Data from the rehabilitation hospitals and the collaborating acute-care study sites will be entered via web-interface of the secure databank. For each value a range is defined, triggering a warning, if the value lies outside the range. A warning also occurs in case data is missing. Volume cloning ensures instantaneous data backup. Pseudonymized data will be stored for a minimum of 10 years to enable data reanalysis and sustained availability.

### Confidentiality

Data acquisition and storage is performed in accordance with the regulatory requirements of General Data Protection Regulation (GDPR) directives. Case report forms or other data that might be photocopied for verification by authorized persons will be pseudonymized, i.e., they will not contain the name of the participants but only their unique identification code. The identification code consists of an abbreviation plus the number of the participant. Published data (e.g., in form of a scientific oral presentations or publications) will only contain sufficiently anonymized data. Participants’ files will be saved using codes in order to protect their privacy.

Plans for collection, laboratory evaluation and storage of biological specimens for genetic or molecular analysis in this trial/future use.

A blood sample of each participant will be collected and stored in the local biobank (Hertie Institute for Clinical Brain Research) to provide a resource for possible further studies to determine fluid biomarkers for stroke outcome. This will be done only in those participants agreeing to an additional broad informed consent for storage and future testing (including possible genetic analyses). The scientists carrying out analyses on these materials will not have access to personal identifiers and will not be able to link the results of these tests to personal identifier information. No individual results will be presented in publications or other reports. Participants will not be informed on an individual basis of any results from these studies.

### Statistical methods

Statistical methods for primary and secondary outcomes.

Only those participants will a complete data set (including the 90 days follow-up examination or a lethal outcome) will be included in the statistical data analysis. Previous studies suggested that the FM-UE endpoints (i.e., the change in FM-UE 90 days after the stroke event compared to the initial FM-UE, see definition under “Outcome”) or the recovery trajectories of FM-UE scores can be clustered into 3 to 5 subgroups [4, 40]. We will first use time series clustering methods such as mixture of Gaussian processes [41] to identify such subgroups, then use the inferred cluster labels to build a classifier using lasso-regularized logistic regression to predict the group membership of the patient as a function of the primary data set and rehabilitation data set detailed in Tables 2, 3. The lasso regularization can automatically select a sparse subset of the significant features, reducing the dimensionality of the feature space and potentially the sample size required. We will use a nested-cross validation setup to tune the regularization strength via the 1SE-rule [29]. We can then use the Gaussian process model of the respective group to predict the primary endpoint and obtain estimates of the fraction of the explained variance of the total variance in the primary end point on data not seen during model training. If the linear model proves insufficient or unsatisfying, we will resort to non-linear regression models such as Generalized Additive Models (GAMs), which allow flexible nonlinear transformations of individual features of pairs of features [42]. Models for secondary endpoints will be built as well.

Interim analyses are not planned since this an observational study without interventions. No further analyses are planned apart from what is described under statistical methods for primary and secondary endpoints. Biobanking of blood samples will allow future (not yet planned) analyses of the relation of blood markers to stroke rehabilitation outcome.

Methods in analysis to handle protocol non-adherence and any statistical methods to handle missing data.

Before model development with participant data will be started, algorithms will be tested with artificial data sets including sets with missing data. Effective algorithms such as nearest neighbors imputation [43] or multivariate imputation [44] on simulated data-sets with missing data will be used as candidate-algorithms for analysis of the empirical data.

Plans to give access to the full protocol, participant level-data and statistical code.

The study protocol is already accessible to the public on the website of the primary study center (https://hih-tuebingen.de/en/research/neurology-and-stroke/research-groups-and-foci/brain-networks-and-plasticity/) [45]. The statistical code will be available in GitHub. Participant-level data can be provided anonymously.

### Oversight and monitoring

#### Composition of the coordinating Centre and trial steering committee

Coordinating center at the University Hospital of Tübingen:

- Conceptual design
- Overall coordination of the study
- Study protocol
- Collection and documentation of the primary data set
- Hosting the databank
- Recruitment of participants

Project partners:

- Collaborating acute-care hospitals in the Centre for Neurovascular Diseases Tübingen:
  ∘ Collection and documentation of the primary data set
  ∘ Recruitment of participants

- Collaborating neurorehabilitation facilities:
  ∘ Collection and documentation of the neurorehabilitation data set

- medical Data Integration Centre (meDIC) at University Hospital of Tübingen:
  ∘ Digitalization of data
  ∘ Programming and maintaining of databank

- Excellence Cluster Machine Learning at the University of Tübingen:
  ∘ Analyzing of data

- Department of neuroradiology:
  ∘ Providing scanner for imaging (MRI and CT)
  ∘ Support in analyzing imaging data

Project management group (including representatives of the University Hospital of Tübingen, the collaborating acute-care hospitals of the Center for Neurovascular Diseases Tübingen, the collaborating rehabilitation facilities, the meDIC, and the Excellence Cluster Machine Learning):

- Collaboration of study sites
- Indicate the direction and promote the project

Task Force data structure (representatives of the University Hospital of Tübingen, the meDIC, the collaborating rehabilitation facilities and the Excellence Cluster Machine Learning):

- Determining the data set
- Digitalization of data
- Data analysis

Task Force Imaging (representatives of coordinating center, and the department of neuroradiology):

- Planning of imaging sequences
- Planning of imaging data processing and analysis

A steering committee or endpoint adjudication committee is not planned given the observational non-interventional design of this study.

#### Data monitoring and reporting of adverse events and harms

Complete and correct data collection and storage will be supervised under responsibility of the principal investigator. Since this an observational study, we do not expect adverse events to occur related to only research procedures and not to usual medical procedures. Nevertheless, adverse events will be collected, reported and assessed using a standardized sheet.

Post-stroke care is provided by the established infrastructure for stroke care. In case of an accident on the way to the hospital or back (for the final examination), a commuting accident insurance is in place.

#### Frequency and plans for auditing trial conduct

The primary study center is affiliated with the Center for Clinical Studies (Zentrum für klinische Studien – ZKS) of the University Hospital Tübingen. This association comes with a yearly external audit as well as regular internal audits (at least two per year) monitoring the accordance of study procedures with the law and internal standard operating procedures following the International Organization of Standardization (ISO) standard 9001.

#### Plans for communicating important protocol amendments to relevant parties (e.g. trial participants, ethical committees)

Any modifications to the study protocol that may have impact on the conduct of the study, potential benefit of the patient or may affect patient safety, including changes of study objectives, study design, patient population, sample sizes, study procedures, or significant administrative aspects require a formal amendment to the protocol. Such amendment will be agreed upon by the principal investigator and the project partners and approved by the responsible ethics committees prior to implementation.

Administrative changes of the protocol are minor corrections and/or clarifications that will have no effect on the way the study is to be conducted. These administrative changes will be agreed upon by the principal investigator and the project partners.

#### Dissemination plans

The results of this study will be disseminated via open-access publications in peer-reviewed journals, and presentations in local, national and international meetings and conferences, exclusively using anonymized data. No other publication restrictions apply.

## Discussion

The primary objective of the present study is to identify markers in the acute phase of stroke predicting recovery of UE motor function and determine the most suitable neurorehabilitative treatment in a given patient based on the acquired data sets using machine learning algorithms.

Although we aim to include the whole spectrum of stroke patients (with a FM-UE ≤ 50 points, indicating a relevant deficit), it is possible that the majority of recruited patients will have relatively mild impairment as the whole test battery and the additional electrophysiological examinations might be viewed as strenuous, particularly for severely affected patients. Thus, the data set might be biased towards less affected patients. However, some of the clinical tests and the neuroimaging are part of the usual stroke work-up anyways, and the additional clinical and electrophysiological tests are designed to be executed with a minimal amount of active participation. Thus, we assume that we will be able to collect sufficient data of severely affected stroke patients, in order to draw generalizable conclusions from the study findings.

Whereas the collection of the primary data set in the stroke units is relatively straightforward, it might be more difficult to encompass the rehabilitation process, which is heavily influenced by motivation of the patient and support by relatives. Therefore, we invested a lot of effort in creating items for documenting these aspects of the rehabilitation process by a questionnaire capturing these potentially influencing factors. Hence, we assume we will be able to gather a comprehensive data set of the rehabilitation process.

## Data Availability

All investigators will have access to the final data set. To ensure conformity to data protection rules, any identifying participant information will be removed from the data.

## Abbreviations

ARAT: Action Research Arm Test
BDI: Beck’s Depression Inventory
BI: Barthel Index
CRP: c-reactive protein
CST: Corticospinal tract
CT: Computed tomography
DILA-S: Diagnostic Instrument for Limb Apraxia
DTABR: (delta+theta)/(alpha+beta) power ratio
DWI: diffusion weighted imaging
EEG: Electroencephalography
EMG: Electromyography
FLAIR: fluid attenuated inversion recovery
FMA: Fugl-Meyer-Assessment
FM-UE: Fugl-Meyer-upper extremity score
fMRI: Functional MRI
GAMs: Generalized Additive Models
GDPR: general data protection regulation
ISO: Organization of Standardization
M1: Primary motor cortex
MEP: Motor evoked potential
MEP +: MEP positive
MEP-: MEP negative
MP-Rage: Magnetic Prepared-Rapid Gradient Echo
MRI: Magnet resonance imaging
mRS: Modified Rankin scale
NIHSS: National Institute of Health Stroke Scale
NPV: Negative predictive value
NT-proBNP: N-terminal proBrain natriuretic peptide
(pd)BSI: (pairwise-derived) brain symmetry index
PCI: Perturbational complexity index
PPV: Positive predictive value
PREP2: Predict Recovery Potential 2
REM: Rapid eye movement
RMT: resting motor threshold
rt-PA: recombinant tissue plasminogen activator
SAFE score: Shoulder abduction finger extension score
SAN: Storage area network
SMA: Supplementary motor area
sMRI: Structural MRI
SS-QOL: Stroke specific Quality of life scale
SU: Stroke Unit
TEP: TMS evoked potential
TMS: Transcranial magnetic stimulation
UE: Upper extremity
